# Compound Danshen Dripping Pill Pretreatment to Prevent Contrast-Induced Nephropathy in Patients with Acute Coronary Syndrome Undergoing Percutaneous Coronary Intervention

**DOI:** 10.1155/2014/256268

**Published:** 2014-10-16

**Authors:** Rong Yang, Liang Chang, Bing-yan Guo, Yan-wei Wang, Ya-ling Wang, Xin Jin, Su-yun Liu, Yong-jun Li

**Affiliations:** Department of Cardiology, Second Hospital of Hebei Medical University, Shijiazhuang 050000, China

## Abstract

*Background*. Contrast-induced nephropathy (CIN) limits the outcome of percutaneous coronary intervention (PCI). *Objective*. To investigate whether pretreatment with Compound Danshen Dripping Pills (CDDP) will decrease the incidence of CIN after PCI. *Methods*. A total of 229 patients with acute coronary syndrome (ACS) undergoing PCI were divided into the control group (*n* = 114) and the CDDP (containing salvia miltiorrhiza and sanqi) group (*n* = 115; given 20 CDDP pills, three times daily before PCI). Serum creatinine, creatinine clearance (CrCl), high-sensitivity C-reactive protein (hsCRP), P-selectin, and intercellular adhesion molecule-1 (ICAM-1) were measured at admission and 24 and 48 h after PCI. *Results*. CrCl decreased after PCI but recovered after 48 h. In the CDDP group, CrCl recovered more rapidly (*P* < 0.05). The procedure increased the hsCRP, P-selectin, and ICAM-1 levels, but these levels were less in the CDDP group (*P* < 0.05). *Conclusions*. Pretreatment with CDDP can decrease the occurrence of CIN in patients undergoing PCI, suggesting that the early use of CDDP is an appropriate adjuvant pharmacological therapy before PCI.

## 1. Introduction

Percutaneous coronary intervention (PCI) is an effective treatment for acute coronary syndrome (ACS). However, contrast-induced nephropathy (CIN) can limit the outcomes of PCI, significantly prolonging hospital stays and increasing mortality [1–5]. The pathogenesis of CIN includes inflammatory mechanisms, endothelial dysfunction, and oxidative stress [6, 7].

Compound Danshen Dripping Pills (CDDP) is a new drug compound containing* salvia miltiorrhiza* and sanqi (*panax notoginseng*). One pill of CDDP (obtained from Tasly Pharmaceutical, Tianjin, China) contains 9 mg of* salvia miltiorrhiza* and 1.76 mg of* panax notoginseng*. Previously, CDDP has been demonstrated to have antioxidant and anti-inflammatory properties, protect endothelial function, inhibit platelet adhesion, and improve microcirculation [8]. We hypothesized that CDDP can reduce the occurrence of CIN. Therefore, a prospective, randomized study was performed to investigate the impact of CDDP on the occurrence of CIN in patients with ACS undergoing PCI. P-selectin, intercellular adhesion molecule-1 (ICAM-1), and high-sensitivity C-reactive protein (hsCRP) can promote cellular and vascular endothelial adhesion, functioning as inflammatory factors, and play a role in thrombosis caused by inflammation. The serum levels of hsCRP, P-selectin, and ICAM-1 were measured before and after PCI to explore the possible mechanisms of action of CDDP.

## 2. Methods

### 2.1. Patients

A total of 367 patients with ACS were treated in the Cardiology Unit of the Second Hospital of Hebei Medical University between January 2012 and February 2013. A total of 236 patients undergoing PCI for ACS were considered for enrollment in this study. ACS was defined as any one of the following: (1) unstable angina pectoris; (2) ST-segment elevation myocardial infarction; and (3) non-ST-segment elevation myocardial infarction. The following exclusion criteria were used: previous contrast media exposure within 7 days of study entry, pregnancy, lactation, renal failure with a creatinine level >3 mg/dL, end-stage renal disease requiring dialysis, history of hypersensitivity to contrast media, multiple myeloma, cardiogenic shock, or left ventricular ejection fraction <40%. Patients who had undergone primary PCI or had undergone PCI within 5 days after enrollment were excluded from the study. The Ethics Committee of the Second Hospital of Hebei Medical University approved this study, which was performed according to Good Clinical Practice standards and the principles of the Declaration of Helsinki and its subsequent amendments. All patients provided written informed consent before enrollment in the study.

Eligible patients were randomly divided into a CDDP group and a control group. Patients were assigned to each study group using an electronic spreadsheet indicating the group assignment by random numbers; randomization blocks were created and distributed. All interventions were performed using standard techniques. All patients received a loading dose of 300 mg of aspirin and 300 mg of clopidogrel at admission. They were hydrated with intravenous isotonic saline (0.9%) at a rate of 1 mL/kg body weight per h for 6–12 h before and 12 h after coronary catheterization to achieve a urinary flow rate of ≥150 mL/h within 6 h after PCI. PCI was performed by the standard Judkins technique, using the right transradial or femoral arterial approach [8]. Intravenous heparin was routinely used during PCI. PCI success was defined as a postprocedural thrombolysis in myocardial infarction grade 3 flow and a decrease of stenosis to <20% residual narrowing by quantitative coronary analysis. The iso-osmolar, nonionic contrast medium iodixanol (Visipaque; GE Healthcare; Carrigtohill, Ireland) was used in patients with chronic kidney disease; and the low-osmolar, iodinated contrast agent iohexol (Iopromide Injection; GE Healthcare; Guangzhou, China) was used in the other patients. Weight and creatinine-adjusted maximum contrast doses were calculated using the following formula: body weight (kg) × 5 mL of serum creatinine. Glycoprotein IIb/IIIa inhibitors were administered at the physician's discretion. During PCI, bivalirudin was used instead of unfractionated heparin in patients considered at high risk of bleeding (>75 years of age, history of previous bleeding, or low body weight). All patients received 100 mg of aspirin/day and 75 mg of clopidogrel/day at least 4 days before PCI. After the procedure, aspirin (100 mg/day) was continued indefinitely, whereas clopidogrel (75 mg/day) was continued for 1 year.

### 2.2. Intervention

Patients in the CDDP group received 20 CDDP pills three times daily from admission to the day before PCI and 10 CDDP pills three times daily for the following 30 days. Blood samples were taken on the day of admission, the day of PCI, and then 24 and 48 h after PCI to measure serum creatinine levels (Cobas 6000; Roche; Mannheim, Germany). For this study, the postprocedure peak value was used. Creatinine clearance (CrCl) was calculated by the Cockcroft-Gault formula: CrCl = ([140 − age] × weight/serum creatinine × 72), with an adjustment for female gender (CrCl female = CrCl × 0.85) [9]. Peripheral venous blood (4 mL) was collected after 10 h of fasting, placed in sodium citrate tubes, and centrifuged at 4°C at 3000 rpm for 10 min to isolate the serum. The hsCRP concentration was determined using the immunoturbidimetric method (Automatic Biochemical Analyzer; Abbott Laboratories; Chicago, IL, USA). Plasma levels of P-selectin and ICAM-1 before and 24 and 48 h after PCI were measured using an ELISA kit (Santa Cruz Biotechnology; Santa Cruz, CA, USA).

The primary end-point was the development of CIN, which was defined as a postprocedure increase in serum creatinine of ≥44.2 *μ*M (0.5 mg/dL) or >25% from baseline [10]. Additional end-points included (1) postprocedural decrease in the estimated glomerular filtration rate of ≥25% at 48 h [11]; (2) postprocedural acute renal failure defined as a rapid decrease in renal glomerular filtration with an increase in baseline creatinine of 176.8 *μ*M (2 mg/dL) [10, 11]; and (3) correlation of hsCRP peak levels after PCI with the occurrence of CIN.

### 2.3. Statistical Analysis

We hypothesized a 15% incidence of CIN in the control group [12] and a 2% incidence in the CDDP group; therefore, a total sample size of 186 patients (93 in each group) would provide 90% power to detect the difference with an alpha level of 0.05. The results are expressed as means ± SD unless otherwise specified. Continuous variables were compared by the *t*-test for normally distributed values; otherwise, the Mann-Whitney *U* test was used. Proportions were compared using Fisher's exact test when the expected frequency was <5; otherwise, the chi-squared test was applied. Analysis of variance was used to compare the different serum creatinine levels and CrCl at baseline and 24 and 48 h after the procedure in each group. *P* values < 0.05 (2-tailed) were considered statistically significant. Analysis was performed using the Statistical Package for Social Sciences, version 10.0, software (SPSS; Chicago, IL, USA).

## 3. Results

A total of 236 patients undergoing PCI for ACS were screened at the Second Hospital of Hebei Medical University from January 2012 to February 2013. Of these patients, seven were excluded due to them having undergone PCI within 5 days. Thus, 229 patients were included in this study and were randomly assigned to two groups: the CDDP group (*n* = 115) and the control group (*n* = 114).

The clinical and procedural characteristics were similar among patients in the two groups (Tables 1 and 2). The mean age, gender distribution, risk factors, and clinical presentations did not differ between the two groups. The preprocedural laboratory results and medications used before the procedure were also not different. The serum creatinine and the corresponding CrCl levels were similar in the two groups before PCI. The incidences of preexisting chronic kidney disease (defined as an estimated glomerular filtration rate < 60 mL/min/1.73 m^2^) were also not different.

**Figure 1:**
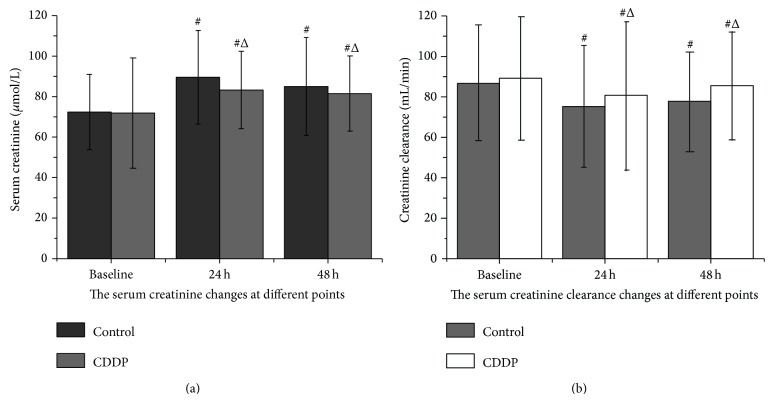
Serum creatinine levels and creatinine clearance before and after PCI.  ^△^ *P* < 0.05, compared with the control group; ^#^ *P* < 0.05, compared with baseline.

For both groups, the serum creatinine level increased significantly by 24 h after the procedure (*P* < 0.05, compared with the baseline level). In the CDDP group at 48 h after PCI, the creatinine level decreased and returned to the baseline level (*P* > 0.05, compared with the baseline level). The creatinine level in the control group, however, failed to decrease significantly at 48 h after PCI (*P* < 0.05; Figure 1). Moreover, the serum creatinine level was less in the CDDP group than in the control group at 24 and 48 h after PCI (*P* < 0.05; Figure 1).

**Figure 2:**
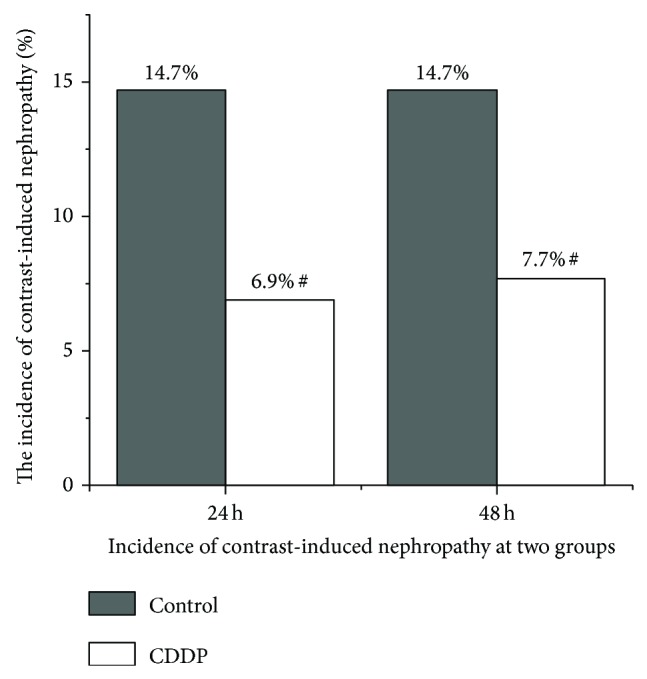
Incidence of contrast-induced nephropathy in CDDP and control patients. ^#^ *P* < 0.05, compared with the control group.

For both groups, the CrCl rate decreased significantly after the procedure, with the lowest value occurring at 24 h and then beginning to increase (*P* < 0.05, compared with the baseline level). At 48 h after procedure, the CrCl rates recovered somewhat but were still significantly less than the baseline level (*P* < 0.05, compared with the baseline level). At this time point, in contrast, the CrCl in the CDDP group increased significantly (*P* < 0.05, compared with the control group; Figure 1). Moreover, the incidence of CIN was significantly less in patients in the CDDP group (*P* < 0.05, compared with the control group; Figure 2) at 24 and 48 h.

**Figure 3:**
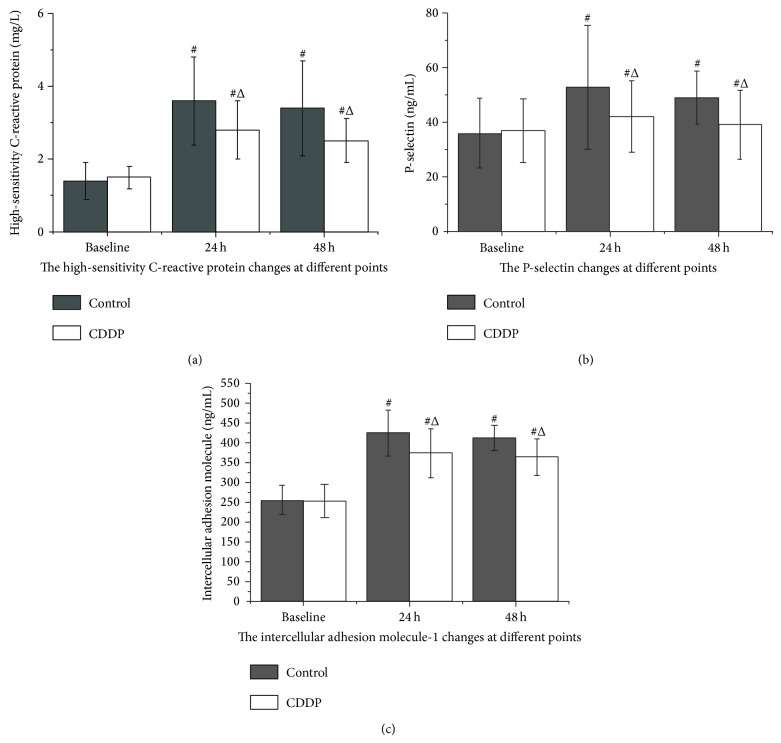
Plasma high-sensitivity C-reactive protein, P-selectin, and ICAM-1 levels before and after PCI, measured by immunoturbidimetry on an autoanalyzer. ^△^ *P* < 0.05, compared with the control group; ^#^ *P* < 0.05, compared with baseline.

Baseline hsCRP, ICAM-1, and P-selectin levels did not differ between the groups, but they increased at 24 and 48 h after procedure (*P* < 0.05, compared with the baseline level). Pretreatment with CDDP decreased hsCRP, ICAM-1, and P-selectin levels at both 24 and 48 h after procedure, compared to those of the control group (*P* < 0.05; Figure 3).

## 4. Discussion

The present study was designed to assess the therapeutic efficacy of CDDP, a new drug compound preparation, in treating CIN, which is associated with PCI in ACS patients. Multiple markers of renal function were measured in the serum from patients who were randomized to either the control or the CDDP group. PCI patients in the control group exhibited clear evidence of renal damage, including increased levels of serum creatinine, decreased CrCl rates, and increased serum levels of hsCRP, ICAM-1, and P-selectin. Pretreatment with CDDP clearly improved renal function after PCI, suggesting that it may be a useful adjuvant in the treatment of ACS.

Although PCI is an effective treatment for ACS, the occurrence of CIN limits the efficacy of PCI. The incidence of CIN differs widely between published studies, depending on the patient risk profile and the prevalence of factors potentially predisposing patients to such complications, such as advanced age, diabetes mellitus, congestive heart failure, and preexisting impairment of renal function [6, 13–15]. Although CIN occurs only in a small proportion of patients undergoing coronary angiography and PCI, it leads to poor short- and long-term clinical outcomes [4, 16, 17]. Thus, the prevention of CIN may improve the clinical outcome during follow-up.

Various mechanisms are involved in the pathogenesis of CIN. Medullary hypoxia occurs shortly after contrast exposure as a result of adenosine production from the macula densa, release of angiotensin, vasopressin, and endothelin, or decreased synthesis of nitric oxide. Other injury processes may occur, including oxidative stress, release of proinflammatory cytokines, and complement activation, with subsequent cytoplasmic vacuolization, necrosis, interstitial inflammation, and tubular obstruction by protein precipitates [6, 7, 18].

Previously published studies have demonstrated several beneficial effects of CDDP. For example, CDDP was found to inhibit oxidative stress and the adhesion of leukocytes onto vascular endothelial cells and to mediate intravascular protection and repair of vascular injury [19]. CDDP can also inhibit mast cell degranulation and protect arteries from pathogen attack. Furthermore, CDDP possesses anti-inflammatory and antioxidant properties, inhibits platelet aggregation, thrombosis, and albumin exudation, and reduces blood viscosity, thereby reducing microcirculation disorders.

Because CIN often occurs within 48 h of contrast medium exposure, it was critical in the experimental design to obtain samples from patients soon after their PCI procedure. The serum creatinine level increased, peaked at 24 h, and then gradually decreased. Compared with the control group, the patients pretreated with CDDP displayed significantly lower peak serum creatinine levels. The level of CrCl decreased significantly after the procedure, with the lowest value occurring at 24 h after intervention and then beginning to increase. In the present study, patients in the CDDP group had a lower incidence of CIN as compared to the control group at 24 and 48 h after PCI.

The control group patients who developed CIN had higher levels of hsCRP, ICAM-1, and P-selectin after PCI, and the reduction of CIN by pretreatment with CDDP was paralleled by a significant decrease in postintervention hsCRP, ICAM-1, and P-selectin levels. These findings suggest that inflammatory mechanisms may be involved in the pathogenesis of CIN and that renal protection is likely to be a result of the anti-inflammatory properties of CDDP. Patients with ACS suffer from excessive inflammation and endothelial dysfunction, ongoing platelet aggregation, cell adhesion, and microcirculation obstruction after PCI. Thus, these patients may gain the greatest benefit from early CDDP therapy before an invasive strategy of renal and myocardial protection is implemented. These results suggest that the early use of CDDP is an appropriate adjuvant pharmacological therapy before percutaneous coronary revascularization.

## Figures and Tables

**Table 1:** Baseline demographics and clinical features of the patient populations in this study.

Characteristic	CDDP (*n* = 115)	Control (*n* = 114)	*P* value
Age (years)	68 ± 6	67 ± 4	0.756
Gender			
Male	51	46	
Female	64	68	
Hypertension (*n*)			
Systolic blood pressure ≥140 mmHg and/or diastolic blood pressure ≥90 mmHg	78	82	0.434
Hypercholesterolemia (>200 mg/dL) (*n*)	20	13	0.207
Diabetes mellitus (*n*)	43	37	0.462
BMI (kg/m^2^)	24.2 ± 1.6	24.8 ± 1.5	0.633
Cigarette smokers (*n*)	25	14	0.061
Chronic kidney disease (*n*)	2	0	0.159
Clinical presentation			
Unstable angina	71	76	0.384
Non-ST-segment elevation myocardial infarction	28	25	0.691
ST-segment elevation myocardial infarction	16	12	0.499
New York Heart Association heart function classification	1.5 ± 1.3	1.8 ± 1.0	0.767
Preprocedural laboratory results			
Baseline serum creatinine (*µ*M)	80.5 ± 18.7	79.9 ± 27.2	0.975
Baseline creatinine clearance (mL/min)	86.8 ± 28.3	89.2 ± 30.4	0.768

**Table 2:** Angiographic and procedural characteristics of patients.

Characteristic	CDDP (*n* = 115)	Control (*n* = 114)	*P* value
Interval from admission to PCI (days)	6.3 ± 1.5	6.5 ± 1.1	0.887
Coronary vessel treated			
Left main	19 (16.5%)	15 (13.2%)	0.474
Left anterior descending	57 (49.6%)	68 (59.7%)	0.125
Left circumflex	25 (21.7%)	20 (17.5%)	0.496
Right	41 (35.7%)	32 (28.1%)	0.078
Type of procedure			
Balloon only (*n*)	6 (5.2%)	7 (6.1%)	0.763
Stent (*n*)	109 (94.8%)	107 (93.9%)	0.763
Stents used per patient	2.25 ± 1.2	2.00 ± 0.8	0.750
Total stent length (mm)	25.3 ± 5.1	24.0 ± 8.5	0.827
Preprocedural antithrombotic therapy			
Glycoprotein IIb/IIIa inhibitors	83 (72.2%)	79 (69.3%)	0.632
Unfractionated heparin	110 (95.7%)	107 (93.9%)	0.543
Bivalirudin	5 (4.3%)	7 (6.1%)	0.543
Total mean contrast volume (mL)	163.3 ± 35.1	183.3 ± 27.2	0.754

## References

[1] Bartholomew B. A., Harjai K. J., Dukkipati S., Boura J. A., Yerkey M. W., Glazier S., Grines C. L., O'Neill W. W. (2004). Impact of nephropathy after percutaneous coronary intervention and a method for risk stratification. *American Journal of Cardiology*.

[2] Sadeghi H. M., Stone G. W., Grines C. L., Mehran R., Dixon S. R., Lansky A. J., Fahy M., Cox D. A., Garcia E., Tcheng J. E., Griffin J. J., Stuckey T. D., Turco M., Carroll J. D. (2003). Impact of renal insufficiency in patients undergoing primary angioplasty for acute myocardial infarction. *Circulation*.

[3] Marenzi G., Lauri G., Assanelli E., Campodonico J., De Metrio M., Marana I., Grazi M., Veglia F., Bartorelli A. L. (2004). Contrast-induced nephropathy in patients undergoing primary angioplasty for acute myocardial infarction. *Journal of the American College of Cardiology*.

[4] Lindsay J., Canos D. A., Apple S., Pinnow E., Aggrey G. K., Pichard A. D. (2004). Causes of acute renal dysfunction after percutaneous coronary intervention and comparison of late mortality rates with postprocedure rise of creatine kinase-MB versus rise of serum creatinine. *The American Journal of Cardiology*.

[5] Dangas G., Iakovou I., Nikolsky E., Aymong E. D., Mintz G. S., Kipshidze N. N., Lansky A. J., Moussa I., Stone G. W., Moses J. W., Leon M. B., Mehran R. (2005). Contrast-induced nephropathy after percutaneous coronary interventions in relation to chronic kidney disease and hemodynamic variables. *The American Journal of Cardiology*.

[6] McCullough P. A. (2008). Contrast-induced acute kidney injury. *Journal of the American College of Cardiology*.

[7] Tumlin J., Stacul F., Adam A., Becker C. R., Davidson C., Lameire N., McCullough P. A. (2006). Pathophysiology of contrast-induced nephropathy. *The American Journal of Cardiology*.

[8] Judkins M. P., Gander M. P. (1974). Prevention of complications of coronary arteriography. *Circulation*.

[9] Szummer K., Lundman P., Jacobson S. H., Lindbäck J., Stenestrand U., Wallentin L., Jernberg T. (2010). Cockcroft-Gault is better than the Modification of Diet in Renal Disease study formula at predicting outcome after a myocardial infarction: Data from the Swedish Web-system for Enhancement and Development of Evidence-based care in Heart disease Evaluated According to Recommended Therapies (SWEDEHEART). *The American Heart Journal*.

[10] Patti G., Nusca A., Chello M., Pasceri V., D'Ambrosio A., Vetrovec G. W., Di Sciascio G. (2008). Usefulness of statin pretreatment to prevent contrast-induced nephropathy and to improve long-term outcome in patients undergoing percutaneous coronary intervention. *The American Journal of Cardiology*.

[11] Briguori C., Airoldi F., D'Andrea D., Bonizzoni E., Morici N., Focaccio A., Michev I., Montorfano M., Carlino M., Cosgrave J., Ricciardelli B., Colombo A. (2007). Renal insufficiency following contrast media administration trial (REMEDIAL): a randomized comparison of 3 preventive strategies. *Circulation*.

[12] Xinwei J., Xianghua F., Jing Z., Xinshun G., Ling X., Weize F., Guozhen H., Yunfa J., Weili W., Shiqiang L. (2009). Comparison of usefulness of simvastatin 20 mg versus 80 mg in preventing contrast-induced nephropathy in patients with acute coronary syndrome undergoing percutaneous coronary intervention. *The American Journal of Cardiology*.

[13] Mehran R., Aymong E. D., Nikolsky E., Lasic Z., Iakovou I., Fahy M., Mintz G. S., Lansky A. J., Moses J. W., Stone G. W., Leon M. B., Dangas G. (2004). A simple risk score for prediction of contrast-induced nephropathy after percutaneous coronary intervention: development and initial validation. *Journal of the American College of Cardiology*.

[14] Gruberg L., Mintz G. S., Mehran R., Dangas G., Lansky A. J., Kent K. M., Pichard A. D., Satler L. F., Leon M. B. (2000). The prognostic implications of further renal function deterioration within 48 h of interventional coronary procedures in patients with pre-existent chronic renal insufficiency. *Journal of the American College of Cardiology*.

[15] Mehran R., Brar S., Dangas G. (2010). Contrast-induced acute kidney injury. Underappreciated or a new marker of cardiovascular mortality?. *Journal of the American College of Cardiology*.

[16] Rihal C. S., Textor S. C., Grill D. E., Berger P. B., Ting H. H., Best P. J., Singh M., Bell M. R., Barsness G. W., Mathew V., Garratt K. N., Holmes D. R. (2002). Incidence and prognostic importance of acute renal failure after percutaneous coronary intervention. *Circulation*.

[17] Lindsay J., Apple S., Pinnow E. E., Gevorkian N., Gruberg L., Satler L. F., Pichard A. D., Kent K. M., Suddath W., Waksman R. (2003). Percutaneous coronary intervention-associated nephropathy foreshadows increased risk of late adverse events in patients with normal baseline serum creatinine. *Catheterization and Cardiovascular Interventions*.

[18] Zhu G. G., Luo R. Z., Guo Z. X. (2007). Advance of cardiotonic pill on inhibiting platelet activation and aggregation. *Chinese Journal of Cardiovascular Medicine*.

[19] Pei F., Fan B. J., Zhao X. Z. (2008). The influence of compound danshen drop pill on coronary microcirculation in myocardial ischemia reperfusion rat. *Chinese Journal of Gerontology*.

